# Attenuated positive and negative symptoms in patients at clinical high-risk for psychosis

**DOI:** 10.1192/j.eurpsy.2022.895

**Published:** 2022-09-01

**Authors:** M. Omelchenko

**Affiliations:** Mental Health Research Centre, Youth Psychiatry, Moscow, Russian Federation

**Keywords:** Attenuated negative symptoms, Clinical high-risk, Attenuated positive symptoms, Early detection

## Abstract

**Introduction:**

The clinical high-risk for psychosis (CHR) is mainly established by the presence of attenuated positive symptoms (APS), but there is evidence of the role of attenuated negative symptoms (ANS) in the development of psychotic spectrum disorders. It is important to establish a link between APS and ANS in patients at CHR in order to improve early detection of psychosis.

**Objectives:**

Establish the relationship between APS and ANS in depressive patients at CHR.

**Methods:**

130 depressive young in-patients at CHR with APS (average age 19.5) and 71 ones with ANS (average age 19.5) were examined. The HDRS scale was used to assess depressive symptoms, the SOPS scale was used to assess APS and ANS, and the SANS scale was used to assess ANS. The results are presented in median values.

**Results:**

No differences were found between two groups in the severity of depressive symptoms on the HDRS scale and CHR symptoms on the SOPS scale (22 vs 23.5 and 45 vs 43 respectively). Statistically valid differences have been established between the groups in the APS severity on the sub-scale of positive symptoms SOPS: 11 and 7 (p 0.001). No differences in the ANS severity on the sub-scale of negative symptoms were detected (17 and 18.5, p=0.207). There were also no differences in the ANS severity on the SANS scale (40 and 47, p=0.163).

**Conclusions:**

It has been established that patients at CHR with APS also have ANS, which may have clinical significance for early detection of psychosis.

**Disclosure:**

No significant relationships.

